# Using the gross motor function measure evolution ratio to compare different dosage of hyperbaric treatment with conventional therapies in children with cerebral palsy – could it end the controversy?

**DOI:** 10.3389/fneur.2024.1347361

**Published:** 2024-03-08

**Authors:** Pierre Marois, Guy Letellier, Mikaël Marois, Laurent Ballaz

**Affiliations:** ^1^Marie Enfant Rehabilitation Center, Sainte-Justine University Hospital Center, Montreal, QC, Canada; ^2^Paediatric Department, Université de Montréal, Montreal, QC, Canada; ^3^Paediatric Rehabilitation Center ESEAN-APF, Nantes, France; ^4^Department of Physical Activity Sciences, Faculté des Sciences, Université du Québec à Montréal, Montreal, QC, Canada

**Keywords:** hyperbaric treatment, cerebral palsy, gross motor function, physical therapy, selective dorsal rhizotomy, hippotherapy, stem cell therapy, botulinum toxin injection

## Abstract

The Gross Motor Function Measure is used in most studies measuring gross motor function in children with cerebral palsy. In many studies, including those evaluating the effect of hyperbaric treatment, the Gross Motor Function Measure variations were potentially misinterpreted because of the lack of control groups. The Gross Motor Function Measure Evolution Ratio (GMFMER) uses historical data from the Gross Motor Function Classification System curves and allows to re-analyze previous published studies which used the Gross Motor Function Measure by considering the natural expected evolution of the Gross Motor Function Measure. As the GMFMER is defined by the ratio between the recorded Gross Motor Function Measure score increase and the expected increase attributed to natural evolution during the duration of the study (natural evolution yields a GMFMER of 1), it becomes easy to assess and compare the efficacy of different treatments.

**Objective:**

The objective of this study is to revisit studies done with different dosage of hyperbaric treatment and to compare the GMFMER measured in these studies with those assessing the effects of various recommended treatments in children with cerebral palsy.

**Methods:**

PubMed Searches were conducted to included studies that used the Gross Motor Function Measure to evaluate the effect of physical therapy, selective dorsal rhizotomy, botulinum toxin injection, hippotherapy, stem cell, or hyperbaric treatment. The GMFMER were computed for each group of the included studies.

**Results:**

Forty-four studies were included, counting 4 studies evaluating the effects of various dosage of hyperbaric treatment in children with cerebral palsy. Since some studies had several arms, the GMFMER has been computed for 69 groups. The average GMFMER for the groups receiving less than 2 h/week of physical therapy was 2.5 ± 1.8 whereas in context of very intensive physical therapy it increased to 10.3 ± 6.1. The GMFMER of stem cell, selective dorsal rhizotomy, hippotherapy, and botulinum toxin treatment was, 6.0 ± 5.9, 6.5 ± 2.0, 13.3 ± 0.6, and 5.0 ± 2.9, respectively. The GMFMER of the groups of children receiving hyperbaric treatment were 28.1 ± 13.0 for hyperbaric oxygen therapy and 29.8 ± 6.8 for hyperbaric air.

**Conclusion:**

The analysis of the included studies with the GMFMER showed that hyperbaric treatment can result in progress of gross motor function more than other recognized treatments in children with cerebral palsy.

## 1 Introduction

Cerebral palsy (CP) is an umbrella term that describes a group of permanent neurological disorders caused by a brain defect or injury that occurred before, during birth or in the first months after birth. It is a non-progressive condition characterized by motor and muscle tone abnormalities and is the most prevalent motor disorder in children as it affects between 1.5 to 4 infants per thousand live births (1). Children with cerebral palsy have impaired muscle control and coordination, leading to problems with walking, balance, as well as fine motor skills (2). Depending on the brain area damaged, it can also affect speech, deglutition, cognition, vision and hearing. Currently, the cornerstone of treatments to improve motor function in children with CP includes rehabilitation therapies, braces, assistive devices, medications, and surgeries (3). These therapeutic approaches aim to help the children in achieving maximum potential in development, motor abilities, function and autonomy as well as preventing and treating secondary conditions such as musculoskeletal deformities.

State of evidence on the different approaches that aim to improve motor function in children with CP has been published and recently updated (3, 4). Among the reviewed therapies, several were reported as being effective and recommended to improve motor function in children with CP. These therapies included physical, pharmacological, and surgical approaches. However, over the past decades, new approaches have been developed with the purpose of improving the brain function and, thereby, the motor function. For some, despite promising results, the level of evidence concerning their efficiency seemed to be insufficient (3). Among those therapies, hyperbaric treatment (HBT) is a medical treatment that involves breathing various concentration of oxygen in a pressurized chamber (5, 6). It is used to treat various conditions, including decompression sickness, wounds that are difficult to heal, and carbon monoxide poisoning. HBT increases delivery of oxygen to the body, which can help to improve the function of damaged cells, reduce inflammation, and promote healing (7). In this review we will consider that HBOT is a HBT which provides pressurized oxygen (100% O2) and HBAT is a HBT which provides pressurized air (21% O2). One of the goals of HBT for cerebral palsy is to increase oxygen supply to the brain, which might improve neurological function (7). Indeed, breathing in a pressurized environment results in higher levels of oxygen dissolved in the blood plasma –breathing just air at 1.3 ATA results in an increase close to 50% of blood oxygen level (7) and thereby enhancing oxygen delivery to tissues throughout the body, including the brain. Moreover, studies have shown that stem cell mobilization is significantly increased following HBT. MacLaughlin et al. (8) reported that an exposition to hyperbaric air at 1.27 ATA generates up to a three-fold increase in circulating stems cells. Finally, HBT has been shown to up- or down-regulated the expression of thousands of genes; the largest clusters of upregulated genes were the anti-inflammatory genes and those that coded for growth and repair hormone, and the largest clusters of downregulated genes were the pro-inflammatory genes and apoptotic genes (9, 10). Together, these mechanisms could stimulate cerebral plasticity and lead to motor function improvement. Until now the evidence supporting the use of HBT for cerebral palsy has remained relatively limited, making it difficult to argue definitive conclusions about its efficacy. Indeed, the studies conducted so far have often been small-scaled or lacked the presence of a control or placebo group. The controversy on the efficacity of HBT in CP is still going on as some studies have wrongly considered mild hyperbaric pressures as a sham treatment for control groups (11, 12). These claims have been increasingly contested as many powerful healing mechanisms are activated even at very low pressure (8, 13–15).

In the last decades, the Gross Motor Function Measure (GMFM) (16), has been the most utilized standardized tool to evaluate gross motor function in children with CP (17, 18). The GMFM is an observational and reliable tool, easily applied by physiotherapists. To date, GMFM is considered as the best clinical standardized tool for measuring change in gross motor function over time in children with CP (19). During childhood, in a context of standard rehabilitation program as done in developed country, the GMFM score is expected to increase before reaching a plateau (20). Hence, to highlight the effect of treatment, the natural expected increase of the GMFM should be taken into account using a control group (21). Due to the heterogeneity of the CP population and ethical issue, controlled studies are hard to implement and most of them, including HBT studies, report GMFM score variation without comparison with a control group (4, 22). Uncontrolled studies generally determine the effects of a therapy based only on the GMFM variation without considering the expected progression of the GMFM due to natural development and standard rehabilitation (20), which depend on the child’s initial age, motor function level, as well as the study duration. From this perspective, Marois et al. (21) created the Gross Motor Function Measure Evolution Ratio (GMFMER) which is the ratio between measured changes and the expected natural evolution (ENE) of the GMFM for a group of children with CP having the same age and disability level. The ENE is computed based on the reference gross motor function classification system (GMFCS) curves of Hanna et al. (20). It is then possible to compare this natural evolution with the GMFM gain observed during the therapy and to compute the GMFMER. This is a novel and more comprehensive method that allows to interpret the results of treatments with higher levels of accuracy when a control group is not included in the study design. It also allows us to reanalyze previous published studies that were using the GMFM as an evaluation tool and to better estimate the effects of those interventions (21).

Many previous studies were misinterpreted because of the lack of comparison groups. In those situations, the GMFMER can provide a more accurate assessment of HBOT impact on motor function in children with CP. This paper aims to use the GMFMER to revisit studies done with different dosages of HBT and to compare these results to studies assessing the effects of recommended treatments for children with CP.

## 2 Materials and methods

Two searches were conducted by two members of the research team (PM, LB) on the electronic databases PubMed from January 1992 until October 2023. On one hand, using the key words “GMFM” AND “cerebral palsy” a systematic analysis was performed to identify studies which used the GMFM to evaluate the impact of standard treatments in children with CP. Studies reporting results from physical therapy (PT), selective dorsal rhizotomy (SDR), Botulinum treatment (BT), stem cell, and hippotherapy treatment were targeted because these treatments are recommended for children with CP (3). Study inclusions were based on the reading of the title, the abstract, and the full paper, as needed to respond to the inclusion criteria. In case of evaluators disagreement or doubt on criteria interpretations, discussion between evaluators was initiated to reach a final decision. All the studies which responded to the following inclusion criteria were included, (i.) English-language full paper, (ii.) evaluate the impact of a longitudinal treatment, (iii) report the average total score of the GMFM before treatment, (iv) report the GMFM increase or the GMFM total score after treatment, (v) report the time interval between evaluations, (vi) report the average age of the group, (vii) the average age of the group superior to 1 and inferior to 8 years old at the pre- and post-treatment evaluation, respectively, (viii) number of children in each group superior to nine, (ix) more than one study reported the effect of the treatment, and (x) group of children mostly presenting gross motor involvement either with quadriplegia or diplegia. Therefore, groups of children having unilateral CP were excluded because the GMFCS does not apply with the same reliability (23). The search strategy was limited to the one described above considering that the aim was to have a valuable representation about the effect of the most standard therapy in children with CP. On the other hand, using the key words “hyperbaric” AND “cerebral palsy,” a second search was performed to identified all the studies which used the GMFM to evaluate the impact of HBT in children with CP. For this search the references list of each included studies was screened to ensure, as much as possible, the inclusion of all the eligible studies. Studies were eligible if they responded to the above-mentioned inclusion criteria and, (xi) receive HBT treatment, therefore the information needed to compute the GMFMER were available for each group of each included study.

The Expected Natural Evolution (ENE) were computed for each included group using the website application http://gmfmer.ca/ (21). The parameters required to compute the ENE were the mean age of the groups, the average GMFM score of the group at the start and after the treatment, as well as the time interval between the pre- and post-treatment evaluations. Then the GMFMER was calculated by dividing the GMFM variation recorded during the study by the ENE. As an example, a GMFMER of 3 would mean that the children receiving a treatment progressed, during the time of the treatment, 3 times more than what he was expected to, considering the natural improvement of the GMFM with standard therapy (20, 21). The GMFMER resulting from the same treatment were reported as mean ± standard deviation.

## 3 Results

**Figure 1:**
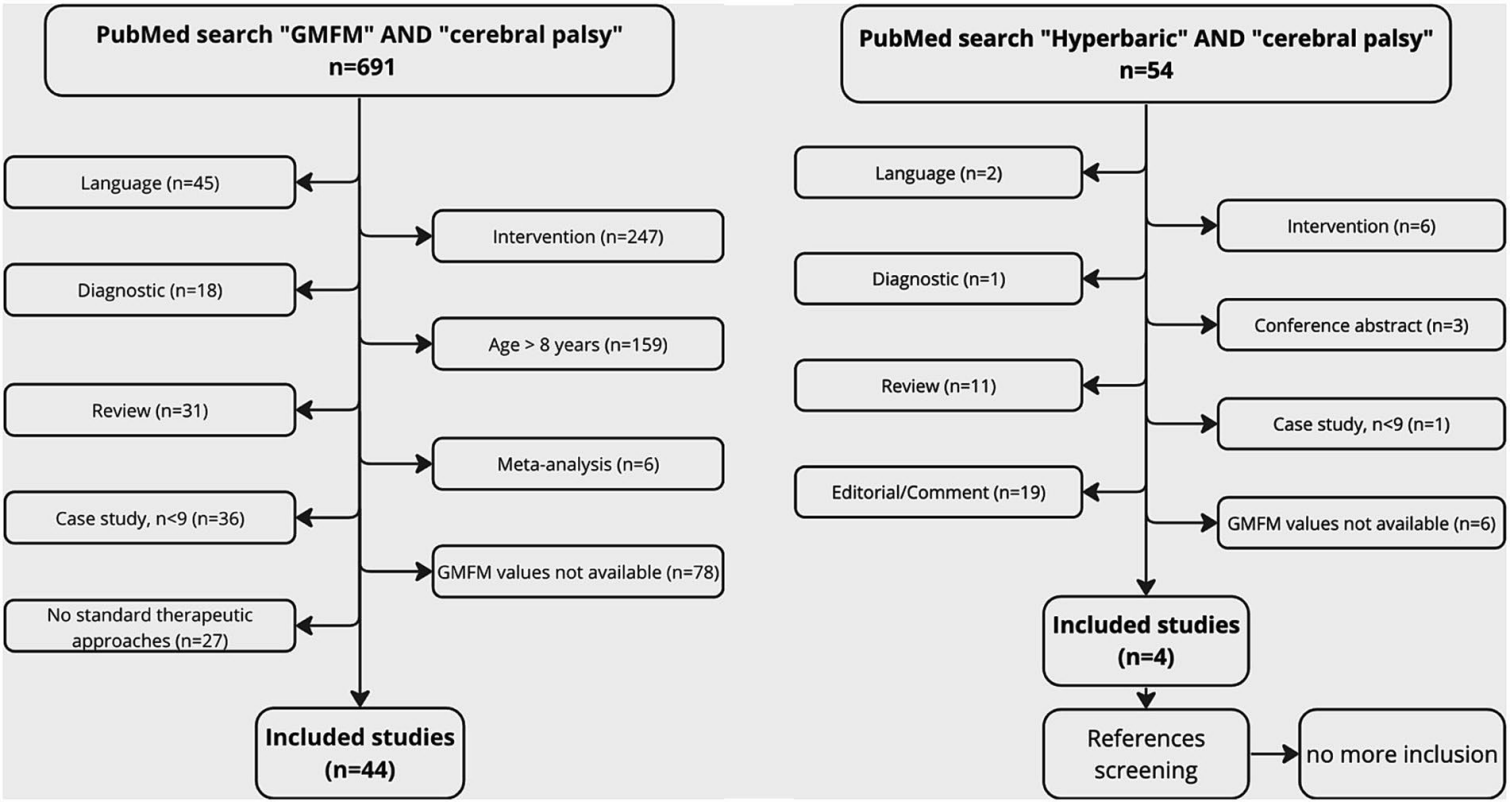
Flow chart of included studies.

The PubMed search using the key words “GMFM” AND “cerebral palsy” gave 691 results. Forty studies (59 groups) responded to the inclusion criteria. The number and the reasons for exclusion were detailed in the studies flowchart (Figure 1). Among the included studies, 24 groups were involved in PT treatment. The intensity of the treatment was characterized based on the time dedicated to therapy per week. Less than 2 h (9 groups), between 2 and 5 h (7 groups), and more than 5 h of treatment (8 groups) were, respectively, defined as common therapy, intensive therapy and very intensive therapy. Groups which were involved in a PT program defined as “usual PT” or “usual care” were pooled with common PT groups. The PubMed search using the key word “hyperbaric” AND “cerebral palsy” gave 54 results. After references screening, 4 studies responded to the inclusion criteria. The number and the reasons for exclusion were also detailed in the studies flowchart (Figure 1). Among the included, 5 groups were treated with HBOT (1.5 or 1.75ATA), 2 groups with HBAT (1.3 ATA), and 1 group with HBT (1.3ATA and 14% O2).

GMFMER of the common PT groups were between 1.1 and 5.7 with a mean of 2.5 ± 1.8. The use of the GMFMER showed that children involved in intense PT (>2 h) improved between 1.8 and 8.6 times more than expected with the natural evolution. Groups of children receiving intensive PT had a GMFMER increase of 4.4 ± 2.7 and children receiving more than 5 h/week of PT had a mean GMFMER of 10.3 ± 6.1 (see Table 1). Rhizotomy produced improvements of GMFMER between 2.4 and 9.5 with an average of 6.5 ± 2.0 for periods up to 12 months. Stem cells treatments resulted in GMFMER between 2.1 and 17.6 with a mean of 6.0 ± 5.9. Mean GMFMER of hippotherapy group (*n* = 2) and BT groups (*n* = 8) were 13.3 ± 0.6 and 5.0 ± 2.9, respectively (see Table 2).

**Table 1:** Physical therapy in children with CP, 24 groups, *n* = 560.

References		Treatments	n	Age (months)	Treatment duration (months)	GMFM initial (%)	GMFM final (%)	GMFM gain	ENE	GMFMER
Sun et al. 2017	(24)	Common PT	31	37.2	12.0	52.0	58.9	6.9	4.4	1.6
Sel et al. 2023	(25)	Common PT	20	56.4	5.3	42.1	42.6	0.5	0.5	1.1
Stark et al. 2016	(26)	Common PT	12	19.4	3.2	32.2	35.5	3.3	2.8	1.2
Kwon et al. 2015	(27)	Common PT	46	70.8	2.0	61.4	61.8	0.4	0.2	2.1
Kwon et al. 2011	(28)	Common PT	16	76.8	2.0	69.8	70.1	0.3	0.2	1.4
Boyd et al. 2001	(29)	Common PT	20	39.0	12.0	40.4	43.2	2.8	2.5	1.1
Trahan et Malouin 1999	(30)	Common PT	24	48.4	8.0	42.1	48.3	6.2	1.1	5.7
Steinbok et al. 1997	(31)	Common PT	69	87.6	12.0	69.0	70.9	1.9	0.6	3.1
Tsorlakis et al. 2004	(32)	Common PT	17	85.2	4.0	65.9	67.1	1.2	0.2	5.2
			Mean	57.9	6.7	52.9	55.4	2.6	1.4	2.5
			SD	23.7	4.4	14.2	13.3	2.5	1.5	1.8
Steinbok, Reiner, et Kestle 1997	(33)	Intensive PT	14	47.0	9.0	62.7	67.8	5.1	2.8	1.8
Wright et al. 1998	(34)	Intensive PT	12	58.3	12.0	56.5	60.9	4.4	1.5	3.0
K. H. Lee et al. 2017	(35)	Intensive PT	24	68.3	9.0	55.3	58.5	3.2	0.7	4.8
Knox et Evans 2002	(36)	Intensive PT	15	88.0	18.0	54.7	55.6	0.9	0.1	6.3
S. H. Lee et al. 2015	(37)	Intensive PT	20	27.6	3.0	32.1	34.4	2.3	1.3	1.8
Sel et al. 2023	(25)	Intensive PT	23	55.2	5.3	40.2	44.2	4.1	0.5	8.6
McLaughlin et al. 1998	(38)	Intensive PT	17	86.4	12.0	71.3	75.5	4.2	0.7	6.0
			Mean	61.5	9.8	53.3	56.7	3.4	1.1	4.4
			SD	21.5	4.9	13.2	13.9	1.4	0.9	2.7
Yi et al. 2013	(39)	Very intensive PT	45	41.0	1.7	48.3	55.5	7.2	0.6	13.0
Polovina et al. 2010	(40)	Very intensive PT	12	17.0	50.5	15.2	52.4	36.8	7.3	5.0
Tsorlakis et al. 2004	(32)	Very intensive PT	17	90.0	4.0	62.2	64.5	2.3	0.2	14.4
Chaturvedi et al. 2013	(41)	Very intensive PT	18	52.8	6.0	44.0	50.0	6.0	0.7	8.5
Huang et al. 2018	(42)	Very intensive PT	27	90.0	24.0	85.0	89.8	4.8	1.8	2.8
Mukherjee et al. 2014	(43)	Very intensive PT	20	42.0	6.0	29.6	32.4	2.8	0.7	3.8
Christy, Chapman, et Murphy 2012	(44)	Very intensive PT	17	91.2	3.0	61.8	64.0	2.2	0.1	18.0
M. Lee et al. 2015	(45)	Very intensive PT	24	28.3	1.0	24.5	29.6	5.1	0.3	17.1
			Mean	56.5	12.0	46.3	54.7	8.4	1.5	10.3
			SD	29.9	17.2	23.1	19.2	11.6	2.4	6.1

ENE, expected natural evolution; GMFM, gross motor function measure; GMFMER, gross motor function measure evolution ratio; PT, physical therapy.

**Table 2:** Commonly used therapy in children with CP, 27 groups, *n* = 986.

References		Treatments	*n*	Age (months)	Treatment duration (months)	GMFM initial (%)	GMFM final (%)	Gain GMFM	ENE	GMFMER
Pennington et al. 2020	(46)	SDR	137	78.5	12.0	59.0	63.6	4.6	0.6	7.5
Wright et al. 1998	(34)	SDR	12	57.8	12.0	51.9	64.0	12.1	1.3	9.5
Law et al. 1997	(47)	SDR	18	51.6	12.0	48.2	57.8	9.6	1.6	6.2
van Schie et al. 2011	(48)	SDR	24	79.0	12.0	56.6	60.9	4.3	0.5	8.0
van Schie et al. 2005	(49)	SDR	9	65.3	12.0	62.8	71.5	8.7	1.3	8.7
Sargut et al. 2021	(50)	SDR	77	72.0	24.0	70.0	77.0	7.0	2.9	2.4
Summers et al. 2019	(51)	SDR	137	72.0	24.0	59.0	66.2	7.2	1.3	5.3
Zhan et al. 2020	(52)	SDR	86	74.4	19.9	53.0	59.1	6.1	0.8	7.4
McLaughlin et al. 1998	(38)	SDR	21	76.8	12.0	70.3	75.2	4.9	1.0	4.9
Nordmark, Jarnlo, et Hägglund 2000	(53)	SDR	18	51.6	12.0	48.2	57.8	9.6	1.6	6.2
Steinbok et al. 1997	(31)	SDR	14	50.0	9.0	60.7	72.0	11.3	2.2	5.2
			Mean	66.3	14.6	58.2	65.9	7.8	1.4	6.5
			SD	11.5	5.3	7.6	7.0	2.7	0.7	2.0
				66.3	14.6	58.2	65.9	7.8	1.4	6.5
Colovic et al. 2012	(54)	BT	16	49.0	6.0	67.8	70.9	3.1	2.1	1.5
Chaturvedi et al. 2013	(41)	PT + BT	18	51.6	6.0	52.0	59.0	7.0	1.03	6.8
Linder et al. 2001	(55)	BT	25	60.0	12.0	54.9	71.1	6.2	1.3	4.9
Moore et al. 2008	(56)	BT	30	63.6	24.0	66.5	71.8	5.3	2.6	2.0
Chang et al. 2017	(57)	BT	71	64.8	5.3	70.5	73.7	3.2	0.9	3.5
Kim, Rha, et Park 2020	(58)	BT	29	85.2	3.5	78.6	81.2	3.5	0.3	10.6
Matsuda et al. 2018	(59)	BT	9	75.6	2.8	62.3	63.3	1.0	0.2	4.6
Polovina et al. 2010	(40)	PT + BT	12	23.0	56.0	20.8	58.9	38.1	6.0	6.3
			Mean	59.1	14.4	59.2	68.7	8.4	1.9	5.0
			SD	18.8	18.2	17.7	7.8	12.1	2.0	2.9
Kwon et al. 2015	(27)	Hippotherapy	45	68.4	2.0	60.8	63.5	2.7	0.2	12.9
Kwon et al. 2011	(28)	Hippotherapy	16	73.2	2.0	70.4	73.7	3.3	0.2	13.8
			Mean	70.8	2.0	65.6	68.6	3.0	0.2	13.3
			SD	3.4	0.0	6.8	7.2	0.4	0.0	0.6
Sun et al. 2022	(60)	Stem cell	23	45.6	12.0	50.3	58.8	8.6	2.4	3.6
Sun et al. 2022	(60)	Stem cell	25	43.2	12.0	48.1	54.7	6.6	2.6	2.6
Sun et al. 2022	(60)	Stem cell	20	43.2	12.0	49.0	58.0	9.0	2.7	3.4
Yousif et al. 2023	(61)	Stem cell	35	51.6	3.0	29.0	32.7	3.7	0.2	17.6
Sun et al. 2017	(24)	Stem cell	32	38.4	12.0	48.9	56.4	7.5	3.6	2.1
Huang et al. 2018	(42)	Stem cell	27	87.6	24.0	85.0	97.7	12.7	1.9	6.6
			Mean	51.6	12.5	51.7	59.7	8.0	2.2	6.0
			SD	18.2	6.7	18.2	21.0	3.0	1.1	5.9

BT, botulinum toxin, ENE, expected natural evolution; GMFM, gross motor function measure; GMFMER, gross motor function measure evolution ratio; PT, physical therapy; SDR, selective dorsal rhizotomy.

**Figure 2:**
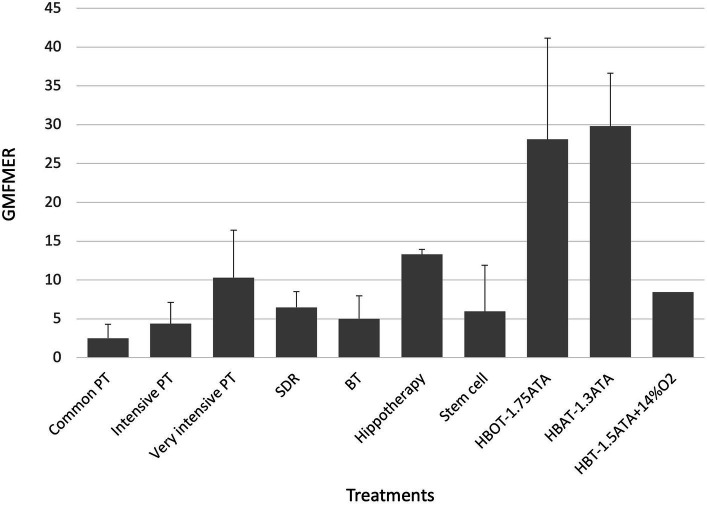
GMFMER for physical therapy, selective dorsal rhizotomy, Botulinum toxin; HBOT, HBAT, HBT, hippotherapy, stem cell therapy. BT, botulinum toxin; GMFMER, gross motor function measure evolution ratio; HBAT, hyperbaric air treatment; HBOT, hyperbaric oxygen treatment; HBT, hyperbaric treatment; PT, physical therapy; SDR, selective dorsal rhizotomy.

Finally, 7 groups from 4 studies reporting the effect of HBOT and HBAT in children with CP were included (see Table 3). The range of the GMFMER was between 16.4 and 47 with a mean of 28.6 ± 11.0. HBT groups were distributed into two subgroups: one composed by the groups (*n* = 5) treated with HBOT at pressures between 1.5 and 1.75 ATA, as the second (*n* = 2) received HBAT at 1.3 ATA. The mean GMFMER of the HBOT and HBAT groups were very similar, 28.1 ± 13.0 and 29.8 ± 6.8, respectively (see Figure 2). Lacey’s “control” group (11) which received a very unusual HBT treatment at 1.5 ATA with just 14% O2, was analyzed separately and had a GMFMER of 8.5.

**Table 3:** HBOT, HBAT and HBT in children with CP, 8 groups, *n* = 316.

References		Treatments	*n*	Age (months)	Treatment duration (months)	GMFM initial (%)	GMFM final (%)	Gain GMFM	ENE	GMFMER
Collet et al. 2001	(62)	HBOT-1.75ATA	57	86.4	2.0	57.3	60.2	2.9	0.1	36.3
Mukherjee et al. 2014	(43)	HBOT-1.75ATA	58	51.6	6.0	32.5	42.1	9.6	0.5	20.9
Mukherjee et al. 2014	(43)	HBOT-1.5ATA	32	51.6	6.0	34.3	42.5	8.2	0.5	16.4
Lacey, Stolfi, et Pilati 2012	(11)	HBOT-1.5ATA	25	75.6	2.0	39.5	40.7	1.2	0.1	20.0
Montgomery et al. 1999	(63)	HBOT-1.75ATA	25	67.2	1.0	56.9	61.6	4.7	0.1	47.0
			Mean	66.5	3.4	44.1	49.4	5.3	0.2	28.1
			SD	15.2	2.4	12.1	10.5	3.5	0.2	13.0
Collet et al. 2001	(62)	HBAT-1.3ATA	54	86.4	2.0	66.3	69.3	3.0	0.1	25.0
Mukherjee et al. 2014	(43)	HBAT-1.3ATA	40	58.8	6.0	29.6	38.6	9.0	0.3	34.6
			Mean	72.6	4.0	48.0	54.0	6.0	0.2	29.8
			SD	19.5	2.8	26.0	21.7	4.2	0.1	6.8
Lacey, Stolfi, et Pilati 2012	(11)	HBT-1.5ATA + 14%O2	25	62.5	2.0	40.7	41.8	1.1	0.1	8.5

ENE, expected natural evolution; GMFM, gross motor function measure; GMFMER, gross motor function measure evolution ratio; HBAT, hyperbaric air treatment; HBOT, hyperbaric oxygen treatment; HBT, hyperbaric treatment.

## 4 Discussion

The present study evaluating the effects of different treatments on children with CP with the GMFMER report original results to estimate the efficacy of recognized or commonly used experimental therapies in children with CP. In total, the GMFMER were calculated for 59 groups. As far as the authors know, previous analysis only considered GMFM improvements without taking into account natural improvement, making it harder to understand the true significance of the measured changes. The GMFMER was established using the GMFCS curves, which were produced using thousands of data points coming from children receiving standard therapy approximately once a week in Ontario, Canada (20). A GMFMER close to 1 is expected in groups of children receiving regular therapy equivalent to standard therapy in Ontario (21). For decades, intensive PT was considered as a powerful method to improve the gross motor skills in children with CP, but the lack of comparison with control groups in many studies implemented with this treatment was often leading to results that were difficult to interpret. Results of the present study showed that PT produce better gains when done more frequently. The GMFMER confirms that intensive PT yields better results, with an average GMFMER of 10.3 obtained for children treated 5 h per week or more (25, 33–38). There is no doubt that SDR is very efficient in decreasing spasticity in children with CP (64) but the true effects on motor function were also difficult to interpret. The results of the present study confirm that this surgery can produce important gains in gross motor function with a mean GMFMER of 6.5 (31, 34, 38, 46–53). In this case the GMFMER value demonstrate a particularly beneficial effect of the treatment, because it applies on a long period, twelve months in most studies (64). Stem cells treatment seems also promising but, as reported with the GMFMER, results are variable in children with CP with GMFMER range between 2.1 and 17.6 (mean, 6.0 ± 5.9) (24, 42, 60, 61). Some studies have reported improvements in motor function (42, 61), while others have shown limited effects with GMFMER values equivalent to those of regular PT (24, 60). Outcomes variability could be due to factors such as cell type, dosage, route of administration, patient age, and severity of cerebral palsy. Since the protocol for this type of treatment can vary tremendously, further analysis would be required to compare the outcomes of the different approaches.

The most important GMFMER value are obtained with HBT. The calculated GMFMER means that children improved their GMFM score on average 28.6 times more than what is naturally expected with standard therapy and 3 times more than with very intensive PT alone (43, 62, 63). The rate of increase of the GMFM score resulting from HBT is also 4 times more than with SDR treatment. Our analysis highlights that the children treated either with HBOT (1.5–1.75 ATA) or with HBAT (1.3ATA) have a very similar progress rate. Furthermore, HBOT and HBAT not only produces impressive gains in gross motor function but studies have also shown significant improvement in fine motor skills, speech, attention and memory (43, 62). The computed GMFMER clearly demonstrates the important change in gross motor skills that HBOT and HBAT can bring to children with CP. The results of the present study corroborates former studies done with people with other neurological conditions including stroke (65), brain trauma (66), autism (67, 68). These studies have also reported significant clinical results, which suggest the potential of this treatment in many types of brain dysfunction. While those results show positive impact on CP and other neurological conditions, these are still to this day not recognized indications for HBT. The most recent meta-analysis on HBT and CP, by Laureau et al. (12), concluded on its inefficacity. In our opinion this analysis was flawed in a multitude of ways. In their analysis, Laureau et al. (12) arrived at non favorable conclusion regarding HBT because of errors in their analysis and interpretation of the included randomized control trials. Indeed, the study published by Collet et al. (62) must be considered as a trial comparing two different dosage of HBT, not as a study comparing HBT to a sham or placebo treatment, as considered by Laureau et al. (12). More specifically, one group was treated with HBOT and the other with HBAT. In their review, Laureau et al. (12) considered the group receiving HBAT as a control and this false assumption completely changes their interpretation of the study. Regarding the physiological effect of even low pressured air and the value of the GMFMER reported in the present study, this group should definitively not be considered as a control one to assess HBOT effect. In fact, both groups in this study had a significant improvement of their gross motor function, as well as their speech, attention, memory, and functional skills (62, 69). These points were rightly noted in the Lancet’s editorial that suggested reinterpretation of Collet et al.’s study (13, 62). Moreover, in 2003, the US Agency for Healthcare Research and Quality (AHRQ) concluded that in Collet’s study “The possibility that pressurized room air had a beneficial effect on the motor function should be considered the leading explanation” when analyzing this study (62, 70).

In addition, Laureau et al. (12) did not use the GMFMER to analyze the studies reviewed in their meta-analysis, however they stated that “the GMFMER should be used as the outcome measure for motor function in children with CP, rather than the GMFM, especially for interventions performed over a long period, like HBT.” If they had computed the GMFMER value of the two treatment groups in Collet’s study, they would have found that they were 36.3 and 25.0 for HBOT and HBAT, respectively. These numbers invalidate the possibility that these changes could be attributed to a participation effect or children natural evolution. It is even more confusing that Laureau et al. (12) wrongly classified HBAT (1.3 ATA) as a control group, while they have classified the same treatment as hyperbaric in their analysis of Mukherjee et al.’s study (12, 43) – it certainly cannot be both. A second major error was made in their analysis of Lacey’s study (11), where once again, a group receiving HBT at 1.5 ATA but breathing only 14% oxygen was used as control group. This lower level of oxygen paired with the 1.5 ATA pressurization has been attempted to replicate the levels of oxygen perfusion that would normally be observed under ambient air. However, pressurization alone induce many physiological changes regardless of oxygenation, and it has been shown repeatedly that many powerful healing mechanisms can be activated even with a limited pressure increase (5, 8, 14, 15). For this reason, it is inaccurate to consider any group receiving HBT as a control, regardless of their levels of oxygenation (8, 71–73). Another critical point regarding the Lacey et al.’s study (11) is the fact that they arrived at a negative conclusion regarding HBT whereas they did not complete their study, which was initially planned for 8 weeks (40 treatments). Surprisingly, they also decided to excluded children who had evidence of neonatal hypoxic–ischemic encephalopathy. Based on an interim analysis the trial was stopped after 2 months because the GMFM increase was inferior to 5 in the HBOT groups. This threshold was arbitrarily chosen and did not correspond to a realistic increase of the GMFM score for the groups of children included in this study. Indeed, by using the average starting age, GMFM score of the groups studied, and computing the expected natural evolution during those 2 months, a GMFM increase of 5 over the course of the study corresponds to a GMFMER of 83.3. No treatment has ever shown such drastic improvements in children with CP. It is very questionable to look at absolute GMFM increase to assess the efficacy of a treatment as this increase is highly dependent on the starting age and duration of the study, which is the exact reason the metric of GMFMER was conceived. With Lacey’s data, i.e., GMFM increase of 1.2 for HBOT group and 1.1 for the HBT (“control”) group, the GMFMER values were 20 and 8.5, respectively. The result of this second group is quite impressive, considering that this group was breathing a mixture of gases with a concentration of only 14% oxygen but with a pressurization of 1.5 ATA (11). It certainly yields to some reflection and suggest that the positive changes obtained with HBT or HBAT could be possibly more related to the pressure (even mild) than the oxygen dose. Finally, Lacey’s study cannot be considered as a proof against HBT in CP. Comments expressing deep concerns about the interpretation and conclusion of this study were already published as a Letter (74).

Studies form Fratantonio et al. (75) or Balestra et al. (76) involving humans give reflection and a better understanding of physiological phenomena to pressure exposure and increase in partial pressure of oxygen. The activation and expression of genes involved in response to ambient air pressure variation could be explained by the “normobaric oxygen paradox.” Indeed, they highlight important adaptive responses triggering signaling cascades leading to better known expressions of antioxidant systems such as transcriptional activation of Hypoxia inducible factors (HIF-1α) and microparticles expressing cell-specific proteins leading to DNA repair in various tissues. Mac Laughlin et al. (8) recently published a paper evaluating the effects of HBAT (at 1.27 ATA) on stem cells mobilization. They showed that endogenously mobilized stem and progenitor cells (SPCs) (CD45dim/CD34+/CD133-) were increased by nearly two-fold following 9 exposures (*p* = 0.02) increasing to three-fold 72-h post completion of the final (10th) exposure (*p* = 0.008) confirming durability. Authors concluded that “stem cells (SPCs) are mobilized, and cytokines are modulated by hyperbaric air. HBA likely is a therapeutic treatment. Previously published research using HBA placebos should be re-evaluated to reflect a dose treatment finding rather than finding a placebo effect.”

## 5 Study limitation

There are several limitations related to this study that should be considered in interpreting the results. First, the studies that we analyzed had all the data needed for the calculation of the GMFMER. They all included GMFM measurements. However, some of the older studies used the GMFM-88 instead of GMFM-66. While these two measures are considered to yield similar results (77), the GMFMER was created using the GMFCS curves developed using data evaluating children with GMFM-66. For this reason, it is possible that GMFMER results computed for therapies using GMFM-88 might vary slightly. Second, the GMFMER cannot be used in all the studies done in CP as other types of pertinent evaluations or scores are also regularly used. Third, there was a wide variability in the qualities of the studies as some were randomized or controlled and others were pilot or observational studies. There was also a wide range in the treatment’s protocol of some of the evaluated therapies, especially with stem cells or PT. Fourth, some of the studies were evaluating the effects of a specific therapy that was associated with a secondary treatment. The best example are the studies on SDR in CP as the children also received regular or intensive PT during the whole follow-up period. Consequently, we have to interpret our data and our analysis with some caution and recognize that in some cases it is the combined effects of therapies that produced improvements. Finally, we also have to bring nuances in the interpretation of the GMFMER results as it evaluates the progress obtained by various therapies over the time of duration of the therapy; in certain cases, that increase is maintained over a period of 6 or more months, as seen with SDR or HBT, while the effect can be less prolonged with other treatments.

## 6 Conclusion

The present study compared the effects of HBT with those of other currently used therapy in CP. The use of GMFMER clearly demonstrates that both HBOT and HBAT lead to gross motor function improvements. Based on the GMFMER these improvements are more important than with any other therapy for children with CP. There is no scientific argument that could bring into question the validity of HBT as a treatment for CP. Our data shows that even HBAT at 1.3 ATA can produce GMFM gains much greater than with standard care and produces the same amount of motor improvements as those obtained at higher pressures. It is therefore scientifically fallacious to use such a treatment for control groups when designing or reviewing studies.

HBAT is a very simple treatment when it is done at these low pressures and can be given in portable softshelled chambers. Considering the benefits of HBAT on gross motor function in children with CP, the use of HBAT combined with recognized therapy for all children with CP should be recommended. Well-designed multicenter trials are still welcomed to determine with more precision the best dosages, frequency of administration and indications for different types and etiologies of CP and possibly other neurological conditions.

## Data availability statement

The original contributions presented in the study are included in the article/supplementary material, further inquiries can be directed to the corresponding author.

## Author contributions

PM: Conceptualization, Formal analysis, Investigation, Methodology, Writing – original draft, Writing – review & editing. GL: Formal analysis, Investigation, Writing – review & editing. MM: Formal analysis, Investigation, Methodology, Writing – review & editing. LB: Formal analysis, Investigation, Writing – review & editing, Conceptualization, Methodology, Writing – original draft.
